# Cancer Stem Cells and Its Role in Angiogenesis and Vasculogenic Mimicry in Gastrointestinal Cancers

**DOI:** 10.3389/fonc.2020.00413

**Published:** 2020-03-31

**Authors:** Erik Lizárraga-Verdugo, Melisa Avendaño-Félix, Mercedes Bermúdez, Rosalio Ramos-Payán, Carlos Pérez-Plasencia, Maribel Aguilar-Medina

**Affiliations:** ^1^Facultad de Ciencias Químico Biológicas, Universidad Autónoma de Sinaloa, Culiacán, Mexico; ^2^Laboratorio de Genómica, Instituto Nacional de Cancerología, Ciudad de México, Mexico

**Keywords:** CSCs, esophageal, gastric, colorectal cancer, angiogenesis, vasculogenic mimicry

## Abstract

Cancer stem cells (CSCs) are able to promote initiation, survival and maintenance of tumor growth and have been involved in gastrointestinal cancers (GICs) such as esophageal, gastric and colorectal. It is well known that blood supply facilitates cancer progression, recurrence, and metastasis. In this regard, tumor-induced angiogenesis begins with expression of pro-angiogenic molecules such as vascular endothelial growth factor (VEGF), which in turn lead to neovascularization and thus to tumor growth. Another pattern of blood supply is called vasculogenic mimicry (VM). It is a reminiscent of the embryonic vascular network and is carried out by CSCs that have the capability of transdifferentiate and form vascular-tube structures in absence of endothelial cells. In this review, we discuss the role of CSCs in angiogenesis and VM, since these mechanisms represent a source of tumor nutrition, oxygenation, metabolic interchange and facilitate metastasis. Identification of CSCs mechanisms involved in angiogenesis and VM could help to address therapeutics for GICs.

## Introduction

Gastrointestinal cancers (GICs) are among the most common malignancies worldwide that mainly include gastric, esophageal and colorectal cancers (1). Treatments for GICs commonly are chemotherapy, radiotherapy, surgery and most recently anti-angiogenic therapy. However, the efficiency of these treatments depends on multiple factors such as cancer staging and resistance to treatment and relapse, which are related to Cancer Stem Cells (CSCs) (2).

In normal and tumoral tissues, vasculature supply the nutrients and oxygen required to maintain homeostasis. Blood vessel formation in the embryo occurs by vasculogenesis, a process that involve *de novo* production of endothelial cells (ECs) (3). On the other hand, the process through which new blood vessels are formed by sprouting and splitting from pre-existing ones is called angiogenesis (4), which is an important cancer hallmark.

Self-renewal of CSCs and initiation of tumor is accompanied by the promotion of angiogenesis, through the secretion of proangiogenic factors such as Vascular Endothelial Growth Factor (VEGF) (5). However, angiogenesis is not the unique source of nutrients and oxygen for tumors (6), given that CSCs are able to transdifferentiate into endothelial-like cells enhancing neovascularization (7). This process, called vasculogenic mimicry (VM), is present in different types of cancers and is responsible of providing a sufficient blood supply to tumor tissues (8). Interestingly, CD133 positive glioma cells express that express VEGF are able to increase vascular density (9) and higher recruitment of endothelial progenitor cells (EPCs) is observed in tumors enriched with CSCs (10).

The aim of this review is to compile recent knowledge of gastrointestinal CSCs and their participation in VM and angiogenesis in order to understand the underlying mechanisms that lead to the development of more effective therapies.

## Gastrointestinal CSCs

Tumors are characterized by cell heterogeneity, according to CSCs theory, which hypothesizes that tumors are driven by a small cell subpopulation with stem cell properties, such as self-renewal and differentiation capacity (11, 12). Also, CSCs promote tumor initiation, growth and proliferation, leading to aberrant growth and slow cycle cell replacement, making them resistant to therapies (13) and are able to move outside of the primary site and metastasize (14).

CSCs were first isolated (CD34^+^CD38^−^) from Acute Myeloid Leukemia (AML) patient samples in late 90s. This small population, was capable to transfer AML from human patients to NOD/SCID mice (15). Since then, surface markers have been used to identify and isolate CSCs in several types of cancers, for instance, CD24, CD44, CD90, CD133, and CD166 for Gastrointestinal CSC, and it was demonstrated that they are generally tissue specific (Table 1) (2).

**Table 1:** Surface markers of gastrointestinal cancers stem cells.

**Tumor type**	**Surface marker**	**Reference**
Gastric cancer	CD44^+^/CD24^+^	(16)
	CD44+/CD54+	(17)
	EpCAM^+^/CD44^+^	(18)
Esophageal cancer	CD44^+^	(19)
	CD44^+^/CD24^−^	(20)
	CD44^+^/ALDH1^high^	(21)
	CD44^+^/ICAM1^+^	(22)
Colon cancer	EpCAM^+^/CD44^+^/CD166^+^	(23)
	CD44v6^+^	(24)
	CD133^+^/CD44^+^/ALDH1^+^	(25)
	CD44^+^/CD24^+^	(26)

Regarding to Esophageal Cancer Stem Cells (ECSCs), they were first isolated from Esophageal Squamous carcinoma cell line (ESCC) using colony morphology criteria (27). Nevertheless, isolation of ECSCs now is performed using CD44 and ALDH1 (19, 28).

CD44 was the first marker used to identify Gastric Cancer (GC) Stem Cells (GCSCs) (29). Moreover, the embryonic markers OCT-4, SOX2, NANOG and the surface maker CD133/Prom1 are highly expressed in GCSCs (30). Interestingly, CD44^+^/CD24^+^ GCSCs subpopulation has shown stem cell properties *in vivo* and *in vitro* (16). Also, EpCAM^+^/CD44^+^ phenotype present stem cell characteristics in GC tissues (18) Besides, isolated CD44^+^/CD54^+^ GCSCs from tumors and peripheral blood, are able to generate tumors both *in vitro* and *in vivo* (17). However, other molecules, such as, CD90, CD71, ABCB1, ABCG2, CD133, ALDH1, and Lgr5 are also considered as potential markers to GCSCs isolation (31–35).

Finally, Colorectal Cancer (CRC) Stem Cells (CRCSCs) were first isolated by CD133 expression, showing tumorigenic capabilities in mice (25, 36). Nevertheless, molecules such as EpCAM^+^/CD44^+^/CD166^+^, ALDH^+^, EphB2^+^, LGR5^+^, and CD44v6^+^ are commonly used to CRCSCs isolation from cell lines (23, 24, 37–39), despite these markers are shared with normal mesenchymal stem cells (MSCs). In this regard, it has been recently reported that Dclk1 discriminates between cancer and normal stem cells in the intestine (40).

### CSCs in Vascular Niche

Vascular niches are key for maintaining the stem phenotype, such as, self-renewal, undifferentiated state and dormancy in normal stem cells (41). In cancer context, neo-vascularization plays an important role during carcinogenesis and metastasis. This process was first described by Scherer in glioblastoma, where the cancer cells growth is possible by the proximity of surrounded blood vessels, now called “cancer vascular niche” (42). Normal stem cells and CSCs primordially growth in vascular niches, due to a perivascular microenvironment. However, cancer vascular niche is rich in abnormal blood vessels, connected and organized with each other in a different pattern from normal vessels (43, 44). These abnormalities are induced by hypoxia, low pH and high interstitial hostile fluid pressure, making a selection of hostile cells that can escape from the tumor through aberrant blood vessels to metastasize (45). Angiogenesis within the tumor mass harbors a variety of host-derived cells, regulated by monocytes Tie-2 expression, fibroblasts, ECs, as well as, innate and adaptive immune cells (46, 47).

## Promotion of Angiogenesis by Gastrointestinal CSCs

Angiogenesis can be divided in two types: sprouting and intussusceptive (48–50). In the first one, ECs proliferate and sprout toward an angiogenic stimulator (e.g., VEGF), forming flat structures called filopodia, producing proteolytic enzymes to enhance angiogenic process (51). On the other hand, intussusceptive angiogenesis is independent of ECs, where an existing vessel is divided into two new vessels only by cellular reorganization (52). Interestingly, neovascularization is an important process to support tumor growth and metastasis; usually, tumors reach a size of ~2 mm in diameter when not fed by neovascularization (53). In this regard, CSCs are able to modify tumoral microenvironment by expressing angiogenic factors in order to enhance tumor neovascularization, contributing finally in their maintenance and proliferation (5).

### Esophageal Cancer

Positive cells to Placental growth factor (PLGF), appear to be CSCs in esophageal cancer and have the capability to release PLGF, promoting cancer metastasis by the activation of MMP9 (54). Besides, CSCs that express PLGF are important due to the promotion (55) or inhibition of tumor angiogenesis depending on its interaction with VEGF (56).

### Gastric Cancer

Bone marrow mesenchymal stem cells (BM-MSCs) are implicated in the promotion of tumor angiogenesis in gastric cancer (GC) since SGC-7901 cells in both, *in vitro* and *in vivo* models, increases VEGF release from tumor cells by the activation ERK1/2 and p38 MAPK pathways, resulting in angiogenesis promotion (57). Moreover, gastric cancer-derived MSCs (GC-MSCs) are also able to promote angiogenesis when interact with BGC-823 and MKN-28 GC cell lines, inducing overexpression of pro-angiogenic factors, such as, VEGF, MIP-2, TGF-β1, IL-6, and IL-8 favoring tube formation (58).

Recently, the Leucine-rich repeat and immunoglobulin-like domain-containing Nogo receptor-interacting protein 2 (LINGO2) a novel gastric cancer stem cell-related marker has been associated with cancer progression (59). In this regard, gastric tumor tissues overexpressing LINGO2 shows elevated expression of the angiogenic marker pVEGFR2 and a blood vessel marker CD34, meanwhile the silencing of LINGO2 in Human Umbilical Vein Endothelial Cells (HUVEC) cells results in inhibition of tube formation, suggesting the involvement of positive-LINGO2 CSCs in angiogenesis (59).

### Colorectal Cancer

CRCSCs are able to initiate vascularization via pericytes by growth promotion (5, 60). Thus, lack of pericytes recruitment impacts negatively in tumor size owing to poor vascular structure (61). This is also correlated to worst prognosis, due to leaky vessels that produces elevated local pressure, enhancing progression and metastasis. Nevertheless, higher vascular density has been associated with recurrence, metastasis and patient mortality (5, 62).

Co-cultivation of CRCSCs and SW620 cells enhances its stemness properties. Also, transplantation of SW48 and MSCs support angiogenesis *in vivo* (63). Additionally, conditioned media (CM) from SW480 cells pre-treated with CRCSCs CM enhances HUVEC tube formation and higher levels of VEGFA expression (63). Besides, BM-MSCs are able to induce angiogenesis, when treated with IFN-γ and TNF-α, by VEGF expression via the HIF-1α signaling pathway (64), meanwhile, IL-8 allows tumor angiogenesis (65).

Participation of CRCSCs in tumor neovascularization has been demonstrated in tumor tissues by CD31/CD133/Lgr5 co-expression (10). Besides, CRC cell lines HCT116 and HT29 spheroid-derived cells are able to co-act with endothelial progenitor cells (EPCs) in order to promote migration and tube formation by secreting VEGF. Meanwhile, EPCs also increases tumorigenesis of CRC cells through angiogenesis (10).

## Signaling Pathways of CSCs in Angiogenesis

**Figure 1:**
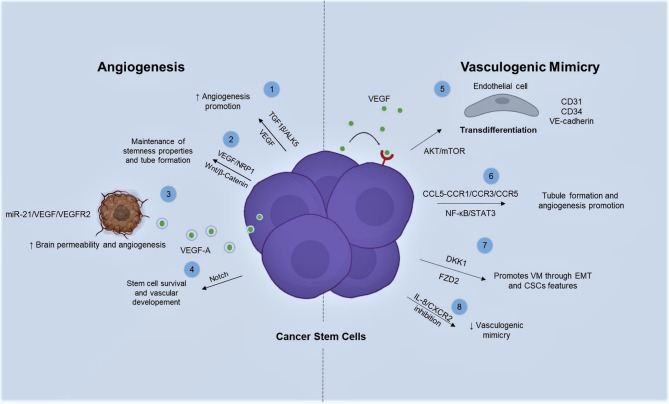
Angiogenic and vasculogenic mimicry promotion by CSCs is mainly triggered by VEGF among several types of cancer. There are different signaling pathways acting in order to promote and sustain neovascularization. 1. Angiogenesis promotion is leaded by TGFβ/ALK5 via VEGF expression in CSCs. 2. Wnt/β-catenin is activated by the interaction of VEGF-A/NRP-1 promoting tube formation. 3. CSCs are able to release VGEF-A by exosomes which in turn stimulates angiogenesis by miR-21/VEGF/VEGFR2 activation. 4. Notch signaling conserves stemness and vasculogenic markers in glioblastoma. 5. VEGFR2 through AKT/mTOR signaling pathway regulates transdifferentiation from poorly differentiated CRC cells into highly expressing CD31, CD34, and VE-cadherin ECs. 6. NF-κB/STAT3 pathway promotes tubule formation and angiogenesis on cancer stem-like cells via CCL5-CCR1/CCR3/CCR5. 7. VM can be influenced by DKK1 by EMT and CSCs behavior. 8. While FZD2 receptor can drive to EMT, thus enhancing stemness properties and VM capabilities.

Little is known about cellular and molecular mechanistic features of CSCs roles in angiogenesis (Figure 1). For instance, Bone Morphogenic Protein 4 (BMP-4) plays a crucial role in angiogenesis by mediating vascular integrity. Besides, VEGF suppression is strongly regulated through BMP-9/ALK1. Conversely, TGFβ1/ALK5 pathway enhances angiogenesis by VEGF expression (66), being a critical signaling molecule for angiogenesis in CSCs (67). Moreover, VEGF-A/NRP-1 interaction promotes stemness properties in breast cancer (BC) cell lines by activation of Wnt/β-catenin pathway, since its inhibition relies in the attenuation of HUVEC-tube formation induced by co-culturing with extracts from Breast Cancer Stem Cells (BCSCs) (60). Moreover, glioblastoma stem-like cells (GSCs) produce VEGF-A, which is secreted in extracellular vesicles promoting permeability and angiogenesis in brain (68). Additionally, angiogenesis promotion can be stimulated by GSC-derived exosomes (GSC-EXs) trough miR-21/VEGF/VEGFR2 axis (69).

Notch signaling pathway is also required for stem cell survival and vascular development and it is a crucial angiogenesis stimulator (70). Interestingly, inhibition of self-renewal capabilities and angiogenesis are orchestrated by Notch signaling repression in GSCs, as well as, reduction of vasculogenic markers, such as, CD105, CD31 and von Willebrand factor (vWF) (71).

## Vasculogenic Mimicry Formation by CSCs in Gastrointestinal Cancers

The generation of vascular channels (VC) without ECs or fibroblasts was first identified in aggressive and metastatic melanoma in 1999, and was termed vasculogenic mimicry (6). In this specific case, the relationship between aggressive melanoma cells that co-expressed Vimentin and epithelial (keratin 8,18) intermediate filaments was particularly interesting, since these cells, where able to be aligned along the external walls of microvascular channels conducing red blood cells, without ECs (72).

Channels formed by VM are composed of a basement membrane and tumor cells that facilitate microcirculation plasma and blood supply from host normal vessels (73). VM can be classified in classical patterns in matrix type (6) and the tubular type (74). Besides, it has been described that VM is composed by matrix proteins such as Laminin, Heparan sulfate proteoglycan, and Collagens IV and VI (75).

VC network may be an independent angiogenesis mechanism for blood source, since angiogenesis inhibitors induce extracellular matrix-rich tubular network formation *in vitro* and are not able to suppress VM in several types of cancers, showing that VM works as an alternative mechanism for blood cells supply (76). Besides, VM is associated with tumor size, short overall survival (OS), high tumor grade, clinical staging, invasion and metastasis (77–79).

Interestingly, tumor cells associated to VM structures acquire an undifferentiated phenotype as well as ECs characteristics (80). Nowadays, CSCs have been involved in VC formation in cancer (81–87). For instance, in salivary adenoid cystic carcinoma (ACC) specimens CD133 is positively associated with VM formation. Besides, CD133^+^ ACC CSCs and xenograft tumors of nude mice injected with these cells show overexpression of VE-Cadherin and VM mediators (MMP-2, MMP-9) (86). Furthermore, an holoclone CD133^+^ isolated from MDA-MB-231 form VM and display MMP-2 and MMP-9 expression (87). In addition, VEGF-silenced cells, attenuate growth and promotes VM as adaptation mechanism associated to HIF-1α expression. Furthermore, enrichment of CD133^+^/CD271^+^ Melanoma CSCs is found in the perivascular niche *in vivo* (81).

### Esophageal Cancer

It has been shown that epithelial–mesenchymal transition (EMT) cells present stem phenotype, showing a remarkable relationship between EMT and CSCs (88). For instances, Ginseng extract showed a negative effect on EMT, as well as, VM in ESCC lines (89). Besides, recombinant Endostatin (rh-Endo) protein combined with radiotherapy downregulates EMT characteristics and VC formation in ESCC through inactivation of AKT/GSK-3β signaling pathway (90).

### Gastric Cancer and Colorectal Cancer

In Gastric adenocarcinoma tissues, a positive relationship between CD133/Lgr5 expression and VC formations, microvessel density, tumor grade, lymph node metastasis and TNM staging has been shown (85). In the case of CRC, the upregulation of ZEB1 results in epithelial phenotype restoration, while, its silencing results in VM inhibition and VE-Cadherin and Flk-1 downregulation in HCT116 cell line (91).

## Signaling Pathways of CSCs in VM

CSCs and VM are involved in cell plasticity, which is the capability of an aberrant population to ECs transdifferentiation (Figure 1) (92). VEGF receptors regulate expression of specific marker for ECs, such as VE-Cadherin (93). In this regard, it has been described that primary and established sarcoma cell lines in contact with post-surgery fluids from Giant cell tumors of bone patients can enrich CD44/CD117 cell population and AKT/mTOR pathway activation. Moreover, it has been proved that prolonged stimulation results in transdifferentiation of tubule-like structures that express endothelial markers, such as, VE-Cadherin and CD31 (94). Additionally, CSCs switch on NF-κB and STAT3 signal pathways via CCL5-CCR1/CCR3/CCR5, stimulating endothelial differentiation and tubule formation (95).

It has been demonstrated that DKK1 enhances VM formation via EMT by developing CSC characteristics in not small cells lung carcinoma (NSCLC) (96). Besides, the Wnt signaling receptor FZD2 drives EMT process, enhancing stem-like properties and VM capacity in HCT116 cells (97). Interestingly, inhibition of IL-8/CXCR2 signaling by Transgelin results in suppression of VM with increased IL-8 levels due to IL-8 uptake inhibition in breast cancer stem cells (BCSCs) (98).

In CRC, the poorly differentiated cell line HCT116 expresses endothelial markers and form tube-like structure *in vitro* after endothelial-conditioned medium co-culture. In addition, under hypoxic conditions cells exhibit higher levels of VEGFR2/VEGFA, as well as, CD31, CD34 and VE-Cadherin overexpression (99).

## Therapeutics Strategies: New Perspectives

Little is known about the role of CSCs promoting angiogenesis and VM. It has been shown that abnormal blood vessels are capable to obstruct immune response to the tumor, as wells as, the transportation and distribution of oxygen and chemotherapeutics. This hostile tumor microenvironment can also lead to selection of cells resistant to radiotherapy and chemotherapy (43). Altogether might suggest that anti-angiogenic drugs often induce tumor hypoxia, allowing CSCs to survive and propagate, thus driving tumor progression.

Nevertheless, some inhibitors of VM are potential molecules to use in therapy of different types of cancers, such as LCS1269 that is capable of overcoming multidrug resistance for DNA-damaging agents in melanoma by VM inhibition (100). In addition, Hinokitiol, a tropolone-associated natural compound, has an important effect over EGFR expression and VM in BCSCs through proteasome-mediated EGFR degradation (101).

Molecules and signal pathways involved in angiogenesis and VM supported by CSCs are novel targets of cancer therapeutics. Nevertheless, information of GICs therapeutics in this matter is limited. Has been described that anti-CD133 has a great potential in treating CRC (96). Besides, targeting signaling pathways is possible, for instance, BBI-608 drug targeting STAT3 could be used for advanced CRC resistant to standard therapeutics or in mixture with Paclitaxel for advanced GC (2, 97). Moreover, Ginsenoside Rg3, a derived from ginseng, represses growth cells and CSCs properties in CRC cells, as well as, inhibits angiogenesis-related genes, suppressing vascularization in xenograft tumors (98).

Several authors suggest that interfering on growth and survival of tumoral ECs can be enough to inhibiting angiogenesis and CSCs self-renewal (99). In this regard, VEGF secreted by cancer cells is a well-recognized therapeutic target and several angiogenic inhibitors have been developed with the capability of also suppress self-renewal of CSCs leading to reduced tumor growth. It has been shown that, Bevacizumab expands survival time by targeting the perivascular niche by the inhibition of VEGF (102). Additionally, bevacizumab reduces metastatic niche formation in rectal carcinoma patients (103) and combined with an anti hepatoma-derived growth factor antibody prevents tumor relapse and progression in NSCLC by impairing CSCs (104). Conversely, the administration of Bevacizumab combined to Sunitinib (VEGF inhibitor) induces tumor hypoxia in BC cell lines resulting in the augment of CSCs population (105).

## Concluding Remarks

Recently, emerging evidence shows that tumors are heterogeneous, being constituted by multiple subpopulations such as CSCs that share self-renewal and differentiation characteristics with normal stem cells. Also, they are able to express specific surface markers that depend on the organ of origin. For instance, CD44, ALDH1, EpCAM, and Lrg5 are characteristics markers of gastrointestinal CSCs, in EC, GC, and CRC. Besides, vascular niches are important for maintaining tumor progression, since CSCs prefer a perivascular microenvironment, rich in blood vessels that often have an abnormal structure and is supported by hostile conditions such as, hypoxia, which in turn, enhances selection of more aggressive cells, able to invade and metastasize. In this regard, CSCs can be transdifferentiated into endothelial-like cells and pericytes, important lineages for maintenance of cancer vascular niche.

Some signaling pathways have been implicated in angiogenesis and VM. The most important molecules and pathways are VEGF/VEGFR2, Notch, BMP9/ALK1, PI3K/AKT/mTOR, NF-κB, and STAT3, that regulate different pivotal processes involved in angiogenesis promotion, such as permeability, endothelial and tubule-like transdifferentiation and promotion of endothelial markers expression, stem cell survival and vascular development.

Clinical relevance of angiogenesis in GICs is remarkable as poor pericyte coverage is correlated with worst prognosis due to leaky vessels that produce elevated local pressure and enhances progression and metastasis. Besides, a higher vascular density in the invasion front has been associated with recurrence, metastasis and patient mortality in CRC. Importantly, Dclk1 can discriminates between cancer and normal stem cells in the intestine.

CSCs are implicated in VM in different cancers, such as ACC, breast cancer and melanoma. In addition, there is a remarkable relationship between EMT and CSCs, due to EMT cells acquired stem phenotype. Importantly, GICs show that the use of drugs, certain proteins or radiotherapy that affect the EMT leads to inhibition of VM. Finally, clinical relevance of VM relies on its association with tumor size, short OS, high tumor grade, clinical staging, invasion and metastasis.

On this front, several drugs have been tested, for instance, Bevacizumab is able to expand survival time by targeting the perivascular niche by the inhibition of VEGF with effect on angiogenesis However, more studies are necessary in order to elucidate CSCs participation on VM and angiogenesis since this could help to address therapeutics for GICs.

## Author Contributions

EL-V, MA-F, MB, and MA-M conceived and designed the content of this review. EL-V, MA-F, MB, and RR-P wrote the paper. CP-P and MA-M contributed to the final version of the manuscript.

### Conflict of Interest

The authors declare that the research was conducted in the absence of any commercial or financial relationships that could be construed as a potential conflict of interest. The handling Editor declared a past co-authorship with several of the authors CP-P, RR-P.
